# Designing Personalized Multimodal Mnemonics With AI: A Medical Student’s Implementation Tutorial

**DOI:** 10.2196/67926

**Published:** 2025-05-08

**Authors:** Noor Elabd, Zafirah Muhammad Rahman, Salma Ibrahim Abu Alinnin, Samiyah Jahan, Luciana Aparecida Campos, Ovidiu Constantin Baltatu

**Affiliations:** 1 College of Medicine, Alfaisal University, PO Box 50927, Riyadh, 11533, Saudi Arabia, 966 554809089; 2 Center of Innovation, Technology, and Education, Anhembi Morumbi University, Sao Jose dos Campos, Brazil

**Keywords:** medical education, personalized learning, prompt engineering, multimodal learning, memory techniques, dual-coding theory, student-centered approach, student-centered, large language model, natural language processing, NLP, machine learning, AI, ChatGPT, medical student, digital literacy, health care professional

## Abstract

**Background:**

Medical education can be challenging for students as they must manage vast amounts of complex information. Traditional mnemonic resources often follow a standardized approach, which may not accommodate diverse learning styles.

**Objective:**

This tutorial presents a student-developed approach to creating personalized multimodal mnemonics (PMMs) using artifical intelligence tools.

**Methods:**

This tutorial demonstrates a structured implementation process using ChatGPT (GPT-4 model) for text mnemonic generation and DALL-E 3 for visual mnemonic creation. We detail the prompt engineering framework, including zero-shot, few-shot, and chain-of-thought prompting techniques. The process involves (1) template development, (2) refinement, (3) personalization, (4) mnemonic specification, and (5) quality control. The implementation time typically ranges from 2 to 5 minutes per concept, with 1 to 3 iterations needed for optimal results.

**Results:**

Through systematic testing across 6 medical concepts, the implementation process achieved an initial success rate of 85%, improving to 95% after refinement. Key challenges included maintaining medical accuracy (addressed through specific terminology in prompts), ensuring visual clarity (improved through anatomical detail specifications), and achieving integration of text and visuals (resolved through structured review protocols). This tutorial provides practical templates, troubleshooting strategies, and quality control measures to address common implementation challenges.

**Conclusions:**

This tutorial offers medical students a practical framework for creating personalized learning tools using artificial intelligence. By following the detailed prompt engineering process and quality control measures, students can efficiently generate customized mnemonics while avoiding common pitfalls. The approach emphasizes human oversight and iterative refinement to ensure medical accuracy and educational value. The elimination of the need for developing separate databases of mnemonics streamlines the learning process.

## Introduction

### Problem Statement

Medical education presents students with the challenge of managing vast amounts of complex information. Mnemonics, memory techniques using associations and patterns, have demonstrated efficacy in improving the encoding and retrieval of medical knowledge [1]. These aids enhance learning and recall by transforming information into more memorable formats through elaborative encoding, retrieval cues, and imagery [2]. However, traditional standardized approaches often fail to accommodate diverse learning preferences, necessitating flexible applications that cater to individual needs.

### Theoretical Framework

Paivio’s dual-coding theory provides the theoretical foundation for this tutorial, supporting the integration of multimodal tools in education. By encoding both verbal and visual information through separate but interconnected pathways, students’ understanding of academic vocabulary can be enhanced [3]. This theory underpins the potential effectiveness of multimodal mnemonics in medical education, particularly when combined with personalization. Research indicates that personalized mnemonic techniques yield superior recall performance compared to standard strategies, with students using self-generated mnemonics demonstrating better performance on recall tasks [4,5].

### Current State of Artificial Intelligence in Medical Education

Recent advances in generative artificial intelligence (AI) technologies have created new opportunities for personalized learning aid creation [6]. AI tools such as ChatGPT and DALL-E have demonstrated proficiency in generating creative and personalized content [7]. ChatGPT, a large language model, uses natural language processing to understand context and generate human-like text responses [8]. It can create diverse textual outputs, including various types of mnemonics. DALL-E, on the other hand, is an AI model designed to generate images from textual descriptions [9]. However, most AI applications in medical education currently focus on data analysis and pattern recognition rather than creative content generation for learning support [10].

### Tutorial Aims and Target Audience

This tutorial aims to provide medical students with practical guidance for creating personalized multimodal mnemonics (PMMs) using AI tools. Through a systematic approach, we detail the step-by-step process of generating text and visual mnemonics using ChatGPT and DALL-E, incorporating templates and examples for effective prompt engineering. The tutorial shares strategies for personalizing mnemonics based on individual learning preferences while addressing common challenges and their solutions in AI-assisted mnemonic creation.

The primary audience includes medical students seeking to enhance their learning through personalized AI-assisted mnemonics. Secondary audiences include medical educators interested in implementing these tools in their teaching practice and students in other health care fields who can adapt these methods to their specific needs. By following this tutorial, readers will learn to create personalized learning aids that combine text and visual elements, potentially improving their ability to retain and recall complex medical information. The approach emphasizes practical implementation while maintaining academic rigor, making it accessible to both novice and experienced users of AI tools in educational settings.

## Methods

### PMMs: Tool Selection and Rationale

This tutorial uses ChatGPT and DALL-E 3 as the primary AI tools for creating PMMs. ChatGPT (GPT-4 model) was selected for text mnemonic generation due to its advanced natural language processing capabilities and ability to generate diverse textual outputs [11]. DALL-E 3 was chosen for visual mnemonic creation based on its proficiency in generating detailed, concept-relevant images from textual descriptions [12]. These tools were selected for their complementary strengths in producing textual and visual content, respectively, allowing for the creation of comprehensive, multimodal mnemonics. Both tools are accessible through OpenAI’s platform, with ChatGPT available through both free and paid subscriptions, and DALL-E 3 using a credit-based system.

### Configuration Settings and Access

For optimal results in medical mnemonic generation, we recommend using ChatGPT’s GPT-4 model with a temperature setting of 0.7, which provides an effective balance between creativity and accuracy in medical content generation. For DALL-E 3, the high-quality setting ensures maximum detail and clarity in visual representations. While both tools offer free tiers, a professional subscription is recommended for consistent access to the latest model versions and enhanced capabilities.

### Prompt Engineering Framework and Quality Assessment

The implementation of PMMs requires a systematic approach to prompt engineering and quality assessment. The process begins with zero-shot learning, where we provide clear instructions without examples, allowing the AI to generate mnemonics based purely on prompt structure. When initial results require refinement, we implement few-shot learning by providing 1 to 2 successful examples to guide the AI. For complex medical concepts, we use chain-of-thought prompting to break down the mnemonic creation process into logical steps.

Zero-shot prompting (providing direct instructions without examples) allows the AI to generate outputs based on its pretrained knowledge [13]. For example, a simple prompt like “Create a memorable mnemonic for the Krebs cycle intermediates” tests the AI’s baseline capabilities without additional guidance.

Few-shot prompting (including 1 to 2 successful examples before the target prompt) helps guide the AI by demonstrating desired outputs [13,14]. For example, showing a successful biochemical pathway mnemonic before requesting one for the Krebs cycle improves output quality by providing clear examples of the expected format and style.

Chain-of-thought prompting breaks complex tasks into logical steps, improving accuracy through structured reasoning [15]. For example, “First, list intermediates. Then, identify key features. Finally, create a mnemonic.” This systematic approach helps ensure comprehensive and accurate outputs, particularly for complex medical concepts.

The implementation followed a five-stage iterative process: (1) template development, in which adaptable prompt templates for text and visual mnemonics are created; (2) refinement, which optimizes prompts through testing of various structures and keywords; (3) personalization, which integrates learning preferences and personal associations and adds options for imagery, humor, and clinical relevance [16]; (4) mnemonic specification, in which prompts for various mnemonic types (acronyms, phrases, rhymes) are created, with corresponding visual representations; and (5), quality control, which is based on peer-to-peer review discussions.

The mnemonics generated through this process were evaluated in peer-to-peer discussions among student authors, facilitated and guided by mentors. This iterative feedback process, integral to quality control, not only ensured medical accuracy and educational value by leveraging student insights and expert guidance, but also trained students to critically assess the quality, accuracy, and effectiveness of AI-generated content. Identified inaccuracies or areas for improvement directly informed subsequent prompt adjustments.

The medical concepts used in this study were carefully selected from ongoing medical courses, ensuring immediate relevance to current learning needs. This selection process focused on identifying complex topics that students found challenging to memorize while ensuring diverse representation of medical subjects and validation against standard medical resources.

For text mnemonic generation, we developed a basic template structure that incorporates medical accuracy, memorability, and personalization elements: “Create a memorable [mnemonic type] for [medical concept]. Focus on [key aspects]. Make it [characteristics: funny/clinical/etc]. Include [specific elements] that relate to [learning context].”

For visual mnemonic creation, the template emphasizes clarity and medical accuracy: “Generate a [style] image depicting [mnemonic content] for [medical concept]. Emphasize [key visual elements]. Ensure medical accuracy and clarity.” These templates serve as starting points and can be customized based on individual learning preferences and specific medical concepts.

### Common Challenges and Solutions

Through our implementation process, we identified several common challenges. Inaccurate medical terminology can be addressed by including specific medical terms in prompts. Unclear visual representations are improved by specifying anatomical or clinical details. When mnemonics become overly complex, requesting step-by-step breakdowns helps maintain clarity and usability. These solutions emerged from practical experience and continue to evolve as we refine the process.

### Ethical Considerations

This educational methodology development did not require formal ethical review as it did not involve human subjects research, collected no personal data, and used only publicly available AI tools as part of regular educational activities.

## Results

The implementation of PMMs using AI tools demonstrated both potential and limitations across a range of medical concepts. Through systematic testing and refinement, we identified key performance metrics, quality assessment outcomes, and practical implementation challenges.

### Generation Performance

The PMM generation process exhibited consistent performance characteristics. Text mnemonic generation via ChatGPT consistently required 2 to 3 minutes per concept. Generating corresponding visual mnemonics with DALL-E 3 required 3 to 5 minutes per concept. Reaching a satisfactory mnemonic typically involved 1 to 3 iterative attempts. The initial success rate, defined as achieving acceptable output on the first attempt, was 85%. After applying quality control and refinement procedures, this success rate increased to 95%. These data suggest that generating PMMs is feasible within a reasonable timeframe, particularly when incorporating iterative refinement.

### Quality Assessment and Examples

Table 1 presents 6 examples of AI-generated PMMs, illustrating the range of mnemonic types generated and highlighting observed successes and areas for improvement. The table includes the target medical concept, the text mnemonic generated by ChatGPT, the visual prompt provided to DALL-E 3, the resulting visual mnemonic, and specific limitations encountered.

Through systematic documentation of the implementation process, we observed that achieving satisfactory results typically required 1 to 3 iterations per concept. Initial attempts often needed refinement in areas such as the precision of medical terminology, visual clarity, or text-image alignment. The examples in Table 1 showcase both successful implementations and instances where refinement was necessary, providing practical insights for users of this tutorial.

The development of a mnemonic for bacterial cell wall antibiotics serves as a representative example. The initial AI-generated mnemonic incorrectly used the term “harbipenems” instead of “carbapenems.” This error, corrected in subsequent iterations, demonstrates the need for human validation, specifically by the medical student or a medical professional. Other examples in Table 1 illustrate the variety of generated mnemonic types, including acronyms, rhymes, and short phrases, paired with their visual counterparts. While generally effective, some visual mnemonics presented challenges in terms of clarity, anatomical accuracy, and alignment with the corresponding text mnemonic.

**Table 1.** Artifical intelligence (AI)–generated personalized multimodal mnemonics.

Medical concept	Prompts used and results	Text mnemonic	Visual and text mnemonic	Observations and limitations
Antibiotics targeting cell wall	First prompt: “Make a mnemonic for antibiotics that target the cell wall, you can group the antibiotics by their mechanism and make it memorable.” Second prompt: “Using the mnemonic, make a memorable photo.” Result: photo was not memorable. Third prompt: “Retry this time make a funny mnemonic.” Result: the cat image.	Mnemonic: “very big purple cats bake happy eggs.” V: vancomycin B: beta-lactams P: penicillins C: cephalosporins B: bacitracin H: harbapenems	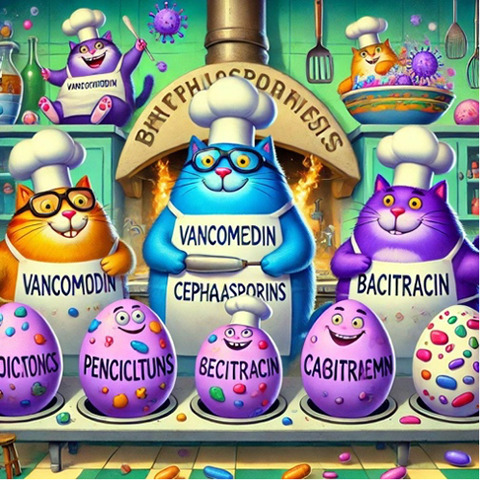	Creative and memorable representation, but incorrect text
Types of leukocytes	Prompt: “Develop a mnemonic to remember the different types of leukocytes. Also, create a funny and cartoonish visual that represents this mnemonic in a memorable way. The illustration should effectively depict the mnemonic.”	Mnemonic (for leukocytes): “never let monkeys eat bananas.” N: neutrophils L: lymphocytes M: monocytes E: eosinophils B: basophils	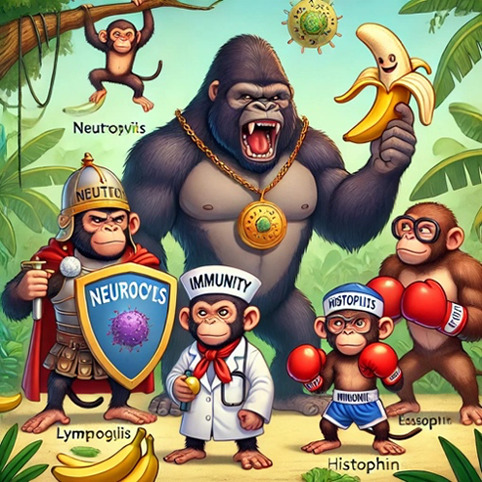	Engaging visualization, but incorrect text
Life cycle of malaria	Prompt: “Create a mnemonic to help remember the malaria life cycle, and design a humorous, cartoon-style visual to represent it in a memorable way. The illustration should clearly depict the mnemonic for easy recall.”	Mnemonic (each word stands for a stage of the malaria parasite’s life cycle): “salty mice bite liver, chilling red blood cells.” S: sporozoites (transmitted via mosquito bite) M: migrate to the liver B: burst out of the liver as merozoites L: liver stages C: chills and fevers (as the parasites enter red blood cells) Red blood cells: parasites invade and replicate in red blood cells	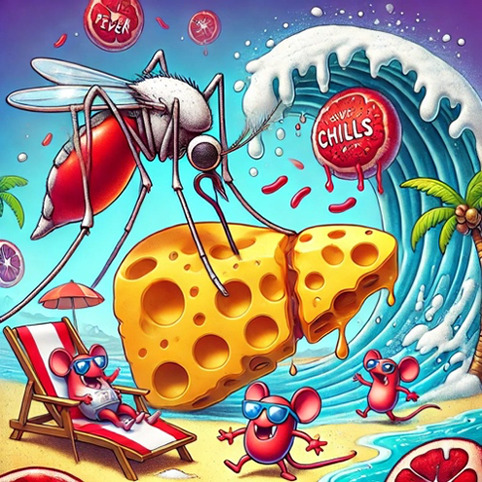	The AI was occasionally unable to fully adhere to detailed prompts in image generation. Additionally, the text included in the image was incorrect.
Renin-angiotensin-aldosterone system (RAAS)	First prompt used: “Generate a funny mnemonic for Renin-Angiotensin-Aldosterone System (RAAS) but make it simple and easy to remember.” Second prompt: “Create simple and memorable image of this mnemonic ‘Keen Elephants Always Raise Blood.’ Use less texts, clear and correct spelling. Place the mnemonic as a title.”	Mnemonic: “keen elephants always raise blood.” K: kidneys release renin when blood pressure or sodium levels are low. E: enzyme (renin) converts angiotensinogen to angiotensin I. A: ACE (angiotensin-converting enzyme) converts angiotensin I to angiotensin II. R: raises blood pressure by vasoconstriction (angiotensin II constricts blood vessels). B: blood volume increases as aldosterone causes sodium and water retention.	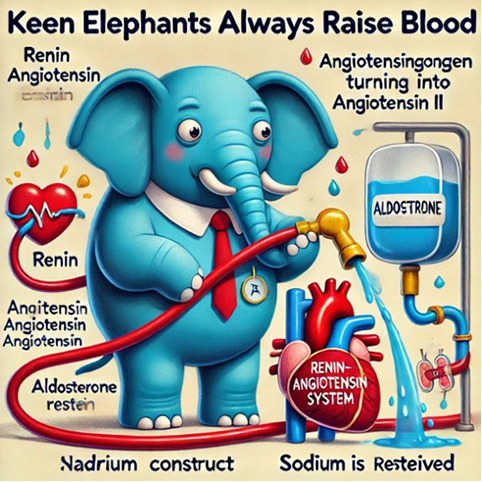	This mnemonic emphasizes the kidney’s keen response to blood pressure regulation. Text is not clear.
Non-selective COX inhibitors	Prompt used: Make a funny memorable picture mnemonic for me to remember the *Non-Selective COX Inhibitors*. They include: Salicylates: Aspirin; Pyrazalone derivatives; Propionic acid derivatives: Ibuprofen, Naproxen; Indole Derivatives: Indomethacin; Anthranilic acid derivative; Aryl acetic acid derivative: Diclofenac; Oxicam derivative; Pyrrole derivatives.”	Mnemonic: “a perfect brew is delightfully aromatic, outstandingly pleasant.” A: aspirin P: pyrazalone derivatives B: ibuprofen I: indole derivatives D: diclofenac A: anthranilic acid derivative O: oxicam derivative P: pyrrole derivatives	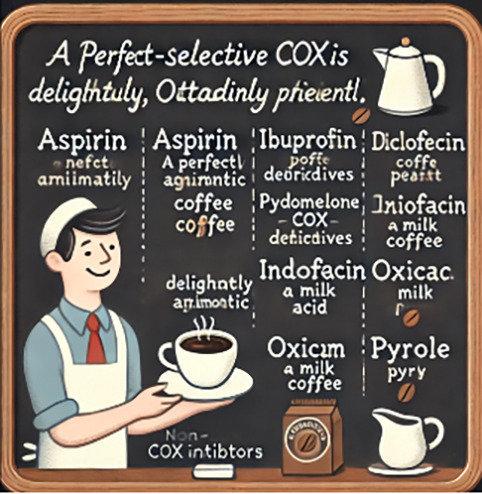	The coffee-themed elements like cups, beans, and mugs may be too abstract and unrelated to the actual topic. The picture lacks clarity and is not memorable.
*Naeglaria fowleri*	First prompt used: “Make an image to remember naeglaria fowleri, brain eating heat loving free living with a mneumonic for its major facts.” Second prompt: “Make it funny: >” Response: “I couldn’t generate the image because the content did not align with the guidelines for image creation. If you’d like, we can adjust the concept or explore a different approach. Let me know how you’d like to proceed!” Prompt: “Try again” Response: “The guidelines for image creation restrict certain content related to how medical or potentially dangerous organisms are portrayed, especially if humor or exaggerated characteristics could be seen as trivializing the topic. Creating a funny depiction of a ‘brain-eating‘ amoeba might fall under those restrictions.”	—^a^		The AI refused to generate the content, highlighting challenges in discerning educational needs from potentially inappropriate content, emphasizing the need for human oversight and refinement.

a Not applicable.

### Generic Templates for Prompt Engineering

For text mnemonic generation (with ChatGPT), the following generic prompt template was developed: “Create a memorable sentence mnemonic for [medical concept]. The mnemonic should be [characteristic 1: funny/simple/easy to remember] and [characteristic 2: relevant to clinical practice/focused on key steps/highlight main components]. Each word or part of the sentence should represent a key aspect of the concept. If possible, incorporate [optional element: wordplay/alliteration/vivid imagery]. Make it relatable to [personal preference: a specific scenario/everyday objects/animals].”

For creation of the visual mnemonic (with DALL-E 3), the following generic prompt template was used: “Generate a [style: cartoon/funny/medical illustration] depicting the mnemonic ‘[text mnemonic]’ for [medical concept]. The image should be [characteristic 1: visually engaging/humorous/clear] and [characteristic 2: memorable/related to the mnemonic words]. Incorporate [specific visual elements: anthropomorphized objects/exaggerated features/relevant symbols]. Ensure any text is minimal, clear, and correctly spelled. Place the mnemonic sentence as a title.”

These templates were iteratively refined based on the quality and relevance of the AI-generated outputs. Examples of specific prompts based on these templates are in Table 1. These templates and examples provide a framework for creating diverse and engaging sentence mnemonics while allowing for customization based on the specific medical concept and desired learning outcomes.

### Implementation Challenges and Refinement Strategies

Three key challenges emerged during implementation, leading to the development of targeted refinement strategies. The first challenge was medical accuracy. Maintaining medical accuracy necessitated continuous verification against established medical resources. Initial outputs occasionally exhibited terminology errors or incomplete conceptual coverage. These issues were addressed by incorporating specific medical terminology in the prompts and implementing a systematic review process involving medical experts.

Second, achieving consistent visual clarity and anatomical accuracy in the AI-generated images presented challenges. Some images lacked clarity or contained inconsistencies between textual and visual elements. We improved visual quality through prompt refinement, including more precise anatomical descriptions and requests for simplified representations of complex concepts.

The third challenge was in content integration. Ensuring seamless integration between the text mnemonic and visual representation required careful prompt design and quality control. A structured review process was implemented to verify that both components effectively reinforced the target medical concept and functioned synergistically to enhance learning.

These findings offer practical observations for educators considering the use of AI-assisted mnemonic generation. While the PMM approach holds promise for personalized learning, our results underscore the essential role of human oversight, domain expertise, and iterative refinement in ensuring accuracy, clarity, and educational value.

## Discussion

### Principal Findings and Implications

This tutorial demonstrates a practical approach to generating PMMs using readily available AI tools. Our findings highlight the feasibility of creating customized mnemonics within a reasonable timeframe (2-5 minutes per concept, with 1-3 iterative attempts). The combination of text and visual elements aligns with dual-coding theory [3,17], potentially enhancing learning and recall. However, challenges related to medical accuracy, visual clarity, and content integration underscore the crucial role of human oversight and domain expertise. The “harbipenems” error, for example, emphasizes the need for medical professionals to validate AI-generated content. These findings suggest that AI-assisted PMM generation can be a valuable tool for personalized learning, but careful attention to quality control and prompt refinement is essential.

### Comparison to the Literature

This tutorial’s approach aligns with the growing interest in applying AI for personalized learning in medical education. While much of the current research focuses on AI for tasks like data analysis [10,18], this tutorial explores the relatively novel application of AI for generating personalized learning content. Our emphasis on multimodal learning resonates with the principles of dual-coding theory [3], which suggests that combining visual and textual representations can enhance learning and memory. Furthermore, the challenges we encountered regarding accuracy and clarity in AI-generated content echo broader concerns in the literature about the need for human oversight in AI-driven educational applications [8,19].

### Strengths and Limitations

This tutorial provides a practical, step-by-step guide for generating PMMs using AI, offering readily adaptable prompt templates and illustrative examples. The student-centered perspective offers valuable insights into the practical challenges and potential benefits of this approach.

This tutorial has several limitations. First, the AI models used may exhibit biases, potentially limiting the diversity and novelty of generated PMMs. Second, inaccuracies in visual representations, such as misspellings or mismatches with the text mnemonic, require careful review and correction. Third, current AI models may refuse to generate content for sensitive medical topics, necessitating alternative strategies or manual content creation. Finally, the lack of a formal evaluation with medical students limits the generalizability of our findings and prevents definitive conclusions about the effectiveness of PMMs on learning outcomes.

### Future Directions

Future research should investigate the effectiveness of PMMs on learning outcomes through controlled studies comparing PMMs to traditional learning methods. Such studies should use objective measures of learning, such as recall accuracy, learning efficiency, and student satisfaction. Further research should also explore the long-term impact of PMMs on knowledge retention and application. The scalability and adaptability of the PMM approach across diverse medical subjects and educational settings warrant investigation. Additionally, future work should address the ethical considerations surrounding AI-generated educational content, including data privacy, bias, and overreliance on technology [8]. Developing guidelines for the ethical and effective use of AI in mnemonic creation and medical education more broadly will be crucial as this field evolves [19].

### Conclusion

This tutorial presents a practical approach to generating PMMs for medical education using the AI tools ChatGPT and DALL-E 3. This approach emphasizes AI as a tool to enhance, rather than replace, traditional learning methods. Originating from medical students seeking to improve their own learning, this tutorial describes a step-by-step process involving prompt engineering, iterative refinement, and quality assessment, illustrated with examples for 6 medical concepts. The personalized nature of the mnemonics, coupled with the multimodal approach, demonstrates potential for enhancing student engagement and facilitating the retention of complex medical concepts. We also highlight key challenges related to medical accuracy, visual clarity, and content integration, underscoring the importance of human oversight and domain expertise in refining AI-generated content. This student-led exploration offers practical guidance and a valuable starting point for educators and students alike interested in leveraging AI for personalized learning in medical education.

## Abbreviations

AI: artificial intelligence
PMM: personalized multimodal mnemonic
